# “*The problem is that our culture is just so messed up about aging.*” Recruiting older men who have sex with men (MSM) into research studies: an example from a study of aging, HIV, and anal HPV.

**DOI:** 10.1186/s12874-022-01752-0

**Published:** 2022-11-18

**Authors:** Alexandra L. Hernandez, Christopher Scott Weatherly, Sahai Burrowes, Jessica Lopez Jimenez, Ryan Gonzalez, Joel M. Palefsky

**Affiliations:** 1 Department of Medicine Division of Infectious Diseases, University of California, San Francisco (UCSF), 513 Parnassus Ave, Room S420, Box 0654, 94143 San Francisco, CA USA; 2 Public Health Program, College of Education and Health Sciences, Touro University, Vallejo, CA USA; 3 College of Osteopathic Medicine, Touro University Nevada, Henderson, NV USA

**Keywords:** MSM, Recruitment, Qualitative, HPV, HIV, Anal Cancer

## Abstract

**Background:**

Anal human papillomavirus (HPV) disproportionately affects men who have sex with men (MSM), particularly those who are older and those living with HIV. After experiencing difficulty recruiting older MSM into a study on aging and anal HPV, we conducted a sub-study to gain feedback on our recruitment methods and explore barriers and facilitators to participating in anal HPV research.

**Methods:**

We conducted focus groups with 30 men who have sex with men (MSM), both HIV-negative and MSM living with HIV, ages 50–75.

**Results:**

We identified multiple themes that were barriers to participation including: (1) *lack of knowledge* about human papillomavirus and anal cancer; (2) *research focused on anal cancer* or discomfort with topics or procedures concerning the anus; (3) *stigma* including stigma associated with being men who have sex with men, being out, being a receptive partner, and being considered “older” in the gay community; and (4) *confidentiality concerns* including a fear of breach of confidentiality. Facilitators to participation were also identified; these *motivational* factors include altruism, wanting recommendations from a doctor, and desire to receive the best available care.

**Conclusion:**

Researchers seeking to enroll older men who have sex with men should be aware of these barriers and facilitators to participation in order to maximize recruitment.

**Supplementary Information:**

The online version contains supplementary material available at 10.1186/s12874-022-01752-0.

## Background

The incidence of anal cancer and associated deaths have been increasing steadily in the United States over the past two decades [1]. Like cervical cancer, anal cancer is causally associated with human papillomavirus (HPV) infection [1]. Men who have sex with men (MSM) are at significantly heightened risk of being diagnosed with anal cancer, and among MSM living with HIV (MSMLWH), the risk is 40 times higher than that of the general population [2].

The Anal HPV, HIV, and Aging Study (AHHA Study) was designed to fill an important gap in knowledge about the prevalence and incidence of anal human papillomavirus (HPV) infection and anal cancer precursor lesions in older MSMLWH and HIV-negative MSM [3]. Almost half of all people living with HIV (PLWH) in the United States (U.S.), including more than 450,000 individuals, are now over the age of 50 [4, 5]. Because the majority of PLWH in the U.S. acquired HIV through sexual contact with another male [6], it can be inferred that there are also a large number of MSMLWH who are over the age of 50. The incidence of several non-AIDS defining illnesses has not decreased with antiretroviral therapy (ART), including anal cancer [5, 7, 8].

The AHHA Study seeks to enroll men or transgender people who have sex with men who are 50 years old or older. The aims of the study include determining the prevalence, incidence, and clearance of type-specific anal HPV infection and anal high-grade squamous intraepithelial lesions (HSIL) by HIV status and age group, as well as examining biomarkers of aging and inflammation [9]. Study visits include a behavioral questionnaire, a grip strength test [10], a 15-foot timed walking test [10], a blood draw, and an anal exam including high resolution anoscopy (HRA)-guided biopsy of visible lesions.

After study initiation, study investigators were immediately challenged by an inability to reach overall recruitment goals. Our target population, MSM ≥ 50 years of age, is a “hard-to-reach” and “hidden” population [11, 12]. Recruitment for the study was well below desired monthly recruitment of approximately 10 newly enrolled participants per month with approximately 2–3 actual enrollments occurring per month, despite using methods that have been successful in recruiting other hard-to-reach populations. These included venue-based sampling [11, 13], referrals from community organizations [14, 15], referrals from clinical providers [14, 15], and advertising in and around medical facilities in the San Francisco Bay Area [14, 15].

To increase enrollment numbers, we conducted a series of focus group discussions (FGD/FGDs) with men who are members of our target population: MSMLWH and HIV-negative MSM ≥ 50 years of age. The main goal of the focus groups was to generate actionable ideas to increase study enrollment. We also sought to identify barriers to participation in our study or studies similar to ours, to identify facilitators participation, and to solicit feedback on recruitment materials developed for the AHHA study.

## Methods

**Fig. 1:**
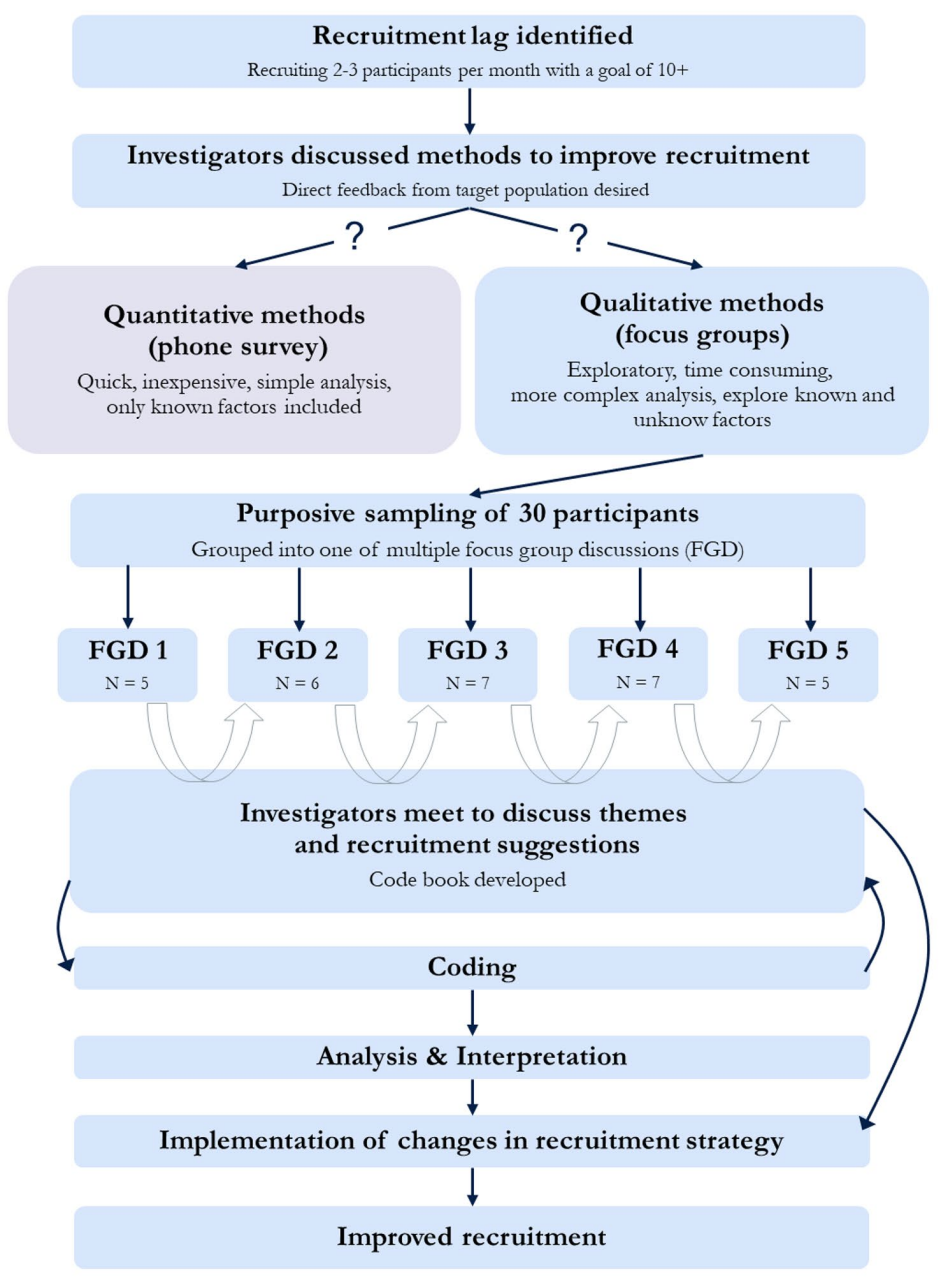
Process flow chart of steps to address recruitment lag via qualitative research study

All methods for this study were approved by the University of California, San Francisco (UCSF) Institutional Review Board before initiation of study procedures. All participants provided written informed consent before any data were collected. We have included a flow-diagram with the process that we followed to complete this study from the identification of a recruitment challenge through to conducting the qualitative study, analysis, interpretation, implementation of changes in recruitment in Fig. 1.

Five FGDs were carried out between June and November of 2018. We asked recruited individuals to answer a few demographic questions on an anonymous paper survey before the beginning of the FGD. Questions were open-ended and the survey requested that participants designate the following characteristics about themselves: age, if they identified as a man or transgender person who has sex with men (or to describe how they identified themselves), their HIV status, and race/ethnicity.

A COnsolidated criteria for REporting Qualitative research (COREQ) checklist [14] was used to ensure quality of the research (Additional file 1). Study methods are described accordingly.

### Participant recruitment

Participants were purposively sampled to achieve balance among the enrollment criteria of HIV status and age group. Each participant had had contact with Anal Neoplasia Clinic, Research and Education Center (ANCRE) clinic. Some FGD participants participated in research in the clinic, some had been screened for the AHHA study, others were research participants in other studies, and some had only been to the clinic as non-research patients. Any potential FGD participant who was a previous research study patient had previously given the ANCRE clinic permission to be contacted for future research studies (documented on informed consent forms). We believed that individuals that had previously interacted with the ANCRE Clinic would likely be willing to come in for a FGD on recruiting for an anal health study and would likely provide the most actionable suggestions. Research team members RG or LC (Table 1) approached each study participant by way of an initial email and followed up with a phone call to schedule the participant into a focus group. Thirty individuals were enrolled in the study and an additional five individuals who were scheduled to come to a FGD did not turn up for their scheduled FGD. All individuals who presented completed the entirety of the FGD; none refused to participate or dropped out. The total number of participants who were contacted and invited to join the study but declined to participate was not recorded.

**Table 1:** Characteristics of Researchers involved in the Focus Group Discussions (COREQ Domain 1, Items 1–5)

Researcher	Role in Focus Groups	Credentials	Occupation	Self-identified Gender	Experience or Training
ALH	Facilitator (FGDs 1–5)	PhD, MPH	Epidemiologist/PI	Cis-female	PI/Trained in qualitative research with SB
CSW	Not present	MPH	Assistant CRC	Cis-male	Trained in study protocol by ALH
JLJ	Not present	BS	Research Assistant	Cis-female	Qualitative methods researcher
JMP	Present (FGD 3)	MD	Medical Doctor/PI	Cis-male	Parent Study PI
LC	Present (FGDs 3–4)	BA	Assistant CRC	Cis-female	Trained in study protocol by ALH
RG	Facilitator (FGDs 1–5)	MA	Senior CRC	Non-binary (presents as male) pronouns “she/her/hers”	Trained in study protocol by ALH
SB	Not present	MALD, PhD	Associate Professor	Cis-female	Qualitative methods researcher

CRC, clinical research coordinator; FGD, focus group discussion; PI, principal investigator

### Focus group procedures

An introductory script containing brief information on HPV, anal cancer, and the AHHA Study was developed and read prior to each FGD (Additional file 2). It contains guidelines for the FGD including maintaining confidentiality, respecting opposing views, and ending on time. A separate focus group guide contains nine open-ended questions for guiding the discussion (Additional file 3). Topics include participants’ knowledge of anal cancer, participants’ views on participating in a study with an anal exam, what may keep other individuals from participating, and what could motivate individuals to participate. Not all questions were asked at each FGD. The focus group guide was not pilot tested but was reviewed by all study researchers.

Five separate FGDs were completed, and each participant completed only one FGD. The focus groups were conducted at UCSF in a private conference room within the building that holds the ANCRE clinic. Participants received lunch or dinner which consisted of a sandwich/salad and drink (<$10) and $25 cash as compensation for their time. FGDs were scheduled for 90 to 120 min in length, and each was completed on time. No one other than the study participants and specified researchers (Tables 1 and 2) were present in the room while any data were being collected. All FGDs were audio-recorded; both RG and ALH additionally took handwritten notes.

**Table 2:** Participant characteristics

Characteristic:		N (%):
**Total #**		30
**HIV Status**		
	Living with HIV	16 (53%)
	HIV-negative	14 (47%)
**Do you identify as** a man who has sex with men?*		
	Yes	28 (93%)
	No*	2 (7%)
**Age**		
	50–59	15 (50%)
	60–69	10 (33%)
	70+	5 (17%)
**Race/Ethnicity**		
	White	21 (70%)
	Latino/Hispanic/Mexican	4 (13%)
	Black or Black Mixed	2 (7%)
	Asian or Asian Mixed	1 (3%)
	Declined to Respond	2 (7%)
**# of Participants per Focus** **Group Discussion (FGD)**, **excluding Study Researchers**		
	FGD 1	5
	FGD 2	6
	FGD 3	7
	FGD 4	7
	FGD 5	5

*Men were asked if they identified as men who have sex with men. If they answered no, they were asked in an open text field to describe their sexual preference. Two participants answered “No” and both described their sexual preference as “bisexual”

### Research team and reflexivity

A description of the researchers involved in the study can be found in Table 1.

Research team members, ALH and RG were present at all FGDs. RG was the primary focus group facilitator and ALH assisted facilitation and clarified questions for participants. RG and ALH introduced themselves at the beginning of each FGD and explained their roles in the study. During FGD 3, the principal investigator of the parent AHHA Study, JMP, was also present and answered a few anal cancer-related questions. During FGDs 3–4, research assistant LC was present but did not contribute to the discussion, otherwise no one else was present for FGDs. The focus group participants were informed that all researchers present at the FGDs were involved in anal cancer and HPV-related research, that they were conducting the AHHA Study itself, and that the AHHA Study was experiencing difficulty recruiting participants.

RG was familiar with most of the study participants and many of the participants knew her to be a clinical research coordinator for several ANCRE studies. ALH knew one participant in FGD 2 as they had worked together in the past. JMP and LC did not know any of the participants in the FGD that they observed. CSW, JLJ, and SB were not present at any of the FGD, and all data were de-identified before they reviewed it.

All researchers involved, including facilitators ALH and RG, are assumed to have a strong interest in the research topics and to have potential biases regarding the importance of the study, anal cancer, and anal cancer screening.

### Data analysis

The study’s methodological orientation was thematic analysis [15]. We employed a mixed, inductive and deductive coding strategy using both *a priori* codes drawn from our research questions and focus group discussion guide, as well as new codes that emerged from the data during analysis (Fig. 1). All themes were developed after focus groups were carried out via analysis during coding.

All FGD audio recordings were professionally transcribed verbatim. Transcripts were read at least once before coding began and read through for coding at least twice by each of our two coders, JLJ and CSW. To ensure consistency in coding, ALH and CSW each coded FGD 1 and reviewed coding results together to ensure consistent coding. CSW and JLJ practiced coding together and subsequently, independently coded and reviewed results together for all five FGDs. New codes and themes were added and updated appropriately during regular coding meetings between CSW and JLJ. Disagreements in coding were brought to ALH and final decisions regarding disagreements were ultimately decided by ALH. Dedoose software was used to apply codes and analyze data [16].

Study investigators determined that saturation had likely been reached when no new themes had emerged from the last focus group [17–19]. After coding, it was confirmed that FGDs 4 and 5 did not yield any original themes. Participants were neither provided with transcripts nor findings subsequent to FGDs.

## Results

### Participant characteristics

Table 2 presents the demographics for the 30 individuals who participated in the five FGDs. FGD had from 5 to 7 participants each. All participants identified as cisgender and are subsequently referred to exclusively as men. Everyone identified as MSM or bisexual, and about half (53%) of the participants were living with HIV. Fifteen men were 50–59 years old, 10 were 60–69 years old, and five were 70+. Each of the five focus groups contained a mix of participants in each of these demographic categories.

The majority of men (68%) described their race/ethnicity as “White” in an open-ended field. Four men described their race/ethnicity as “Latino”, “Mexican/White”, or “Hispanic/White”. Two men described their race/ethnicity as “Black” or “Black Mix”. One person described their race/ethnicity as “Asian/White”.

### Thematic analysis

Table 3 presents each theme and sub-theme identified by the study including a description, example quote, and the number of times the code occurred in each focus group.

**Table 3:** Barriers identified from thematic analysis of focus group transcripts

THEME			Theme frequency by Focus Group Discussion (FGD)																			
**Sub-Theme**	**Description**	**Example Quote**	**1**		**2**				**3**					**4**					**5**			**Totals**
**LACK OF KNOWLEDGE ABOUT HPV/ANAL CANCER**																						
Lack of Knowledge of HPV/Anal Cancer	Expressing a lack of knowledge about anal cancer, anal HPV, anal screening, warts, or HPV vaccination.	“Part of it is not feeling confident that the provider really is able to give me substantial information about my health and sort of, so I feel like have a lot of confusion, have interest about anal cancer and HPV, but I feel like I haven’t really had clear sources of what tests can be done and what information can be gotten from those tests.” *–FGD 1, Participant 3*	10		8						6					11					4	39
**RESEARCH FOCUSES ON ANAL CANCER**																						
Physical Anal Discomfort	Associating the anal exam with physical discomfort.	“Throughout my life I’ve known a lot of gay men that don’t even like their butt touched so. I mean I don’t think it … would be easier [for a gay man]. You’d think, of course, I … think, you know, that [it] would be easier for a gay men because that is the kind [of] activity [they] have, but I know a lot of gay men who don’t like their butt touched, so I know they wouldn’t have this test done.” *–FGD 2, Participant 2*	6		5				2					3					3			19
Discomfort Talking About “Anal” or an Anal Exam	Feeling discomfort regarding the discussion of the anus or the anal exam.	“Well, one thing I am just sort of comparing it to in mind is the way people talk about colonoscopies, and part of it was just the preparation for it, but there are some people who say, oh yeah, it wasn’t really that big a deal. What’s all the fuss about? But a lot of people, they sort of love to dread it. And it is funny because we’re talking about how people don’t like to talk about anal stuff, but people love to talk about colonoscopies, but it’s always about how awful it is, so I don’t hear people talking about anal pap smears that way.” *–FGD 1, Participant 1*	11		3				2					2					2			20
Anus as a Center for Pleasure	Not wanting to associate anus with cancer or disease.	“That’s the thing about this, you know it is talking about disease around the part of your body that you derive pleasure from, and I guess maybe that’s somewhat difficult too when talking about this, you know, first of all talking to myself, talking to friends about anal pleasure and that, and then having a provider/clinician that will listen to me and be okay with that and then talk about the disease layered on top of that as well, this gives me pleasure, but now I have to deal with the fact that this disease could be there…” *–FGD 1, Participant 1*	3		0				0					0					0			3
Location Barrier	Not wanting to think about HPV, anal cancer, or disease at places like bars or while on vacation.	“If I was at the bar, I don’t want to think about all the consequences, my focus isn’t in on health, and then there is other times [like] when I am in my doctor’s office, and I’m thinking a lot about health and things I want to do, to stay healthy, so I think the bar, you will get the message out [to] people so from a familiarity standpoint you will get it, but from a call to action, I think it’s a mental shift the people aren’t going to make a lot to like ‘oh, well now I will go sign up and do this on my phone.’ It is something like who wants to sign up for STD education when you are in a bar, it is like, I don’t want to think about that, I am hoping to meet [someone].” *–FGD 3, Participant 3*	1		0				5					2					3			11
Female Provider	Not wanting a female medical provider to perform the anal exam.	“Yeah, I am not going to discuss my butt with a woman, you know. It is just how I am.” *–FGD 3, Participant 2*	0		3				1					0					0			4
**STIGMA-RELATED BARRIERS**																						
Age Stigma	Expressing judgement in relation to older individuals.	“The problem is that our culture is just so messed up about aging.” *–FGD 1, Participant 4*	3		3		5					0					3					14
Anal STD Stigma	Noting judgment in relation to anal STDs/STIs, including HPV.	“Yeah, and I think for our generation, at least, I know through my life, sort of, anal STDs have more shame than say other STDs.” *–FGD 1, Participant 1*	1		3		2					1					0					7
Stigma Against Being Receptive/Bottom Partner	Expressing a negative association regarding being the receptive sexual partner.	“Like there used to be an assumption that only bottoms get HIV. We have those biases and prejudices, they are not rational, and they are not true, but I think there is still some bottom shaming in our culture.” *–FGD 1, Participant 3*	2		1		0					0					1					4
General Cancer Stigma	Expressing negative thoughts regarding having, getting screened for, or being at risk for any type of cancer, other than anal cancer.	“… But now I have to deal with the fact that this disease could be there, and it is similar to HIV but, you know, it’s very… it’s different enough because then the cancer thing is that the ‘C word’ is used. So that’s just a facet of this that I find maybe prohibitive, and it prohibits discussion and awareness and that sort of thing.” *–FGD 1, Participant 3*	2		1		0					1					0					4
Anal Cancer Stigma	Expressing negative thoughts regarding having, getting screened for, or being at risk for anal cancer.	“With the colonoscopy, they go deeper throughout the whole and entire canal whereas the study just focuses on the first few inches … but still, when you attach anything to the word ‘Cancer’ it is stressful and it is scary and it turns people inverted, some people don’t like to tell their families or their friends because … they get treated differently or something like that, so I think private, I mean you want to put the word out there, but you also want to say it is something that a lot of people maybe want to keep private.” *–FGD 3, Participant 4*	1		0		1					1					0					3
HIV Stigma	Expressing judgment related to having HIV/AIDs.	“… He passed away with HIV and he was the one that was a world traveler, he was all over the place so it stunning to me he was such a world traveler and … he died in a time after the medication was [already available] that could have saved his life. So what happened? And I kept hearing he never got tested [for HIV].” *–FGD 5, Participant 5*	1		2		0					0					8					11
Stigma Against Being Gay/Bisexual/MSM	Expressing a negative association regarding men who have sex with men.	“How many people, do you think are radically in the closet about this stuff … I mean men that have sex with men, they are really underground about it.” *–FGD 5, Participant 5*	0		0		1					5					5					11
**CONFIDENTIALITY CONCERNS**																						
Confidentiality	Concern over the sharing of personal and/or medical information.	“I mean I am familiar with HIV studies and also thinking back to the 80s when anonymity and confidentiality were—you know people were afraid of their providers having records that in some situations, sort of … the idea of preexisting conditions or the ability to sort of be stone-walled out of health coverage is certainly an experience within our memorable history that did not trust that sort of integration.” *–FGD 1, Participant 1*	9		1		6					2					8					26
**GENERAL BARRIERS NOT SPECIFIC TO ANAL CANCER**																						
Insurance Coverage	Concern over coverage of visit costs.	“It might be easier to get people if they didn’t have to pay or figure that if for some reason their insurance declines it, then they’re on the hook for it.” *–FGD 4, Participant 3*	6		6				4					5					1			22
Money as a Barrier	Money concerns related to joining the study, including cost or compensation.	“To me it would be a problem, I mean because I only have my social security return in income so anything that will mean that I have to pay, something, $70 is a lot of money, couple of thousands is just like you might as well kill me.” *–FGD 5, Participant 2*	2		5				1					3					1			12
Time Barrier	Expressing lack of time to be in the study.	“I was also thinking our lives are busy, but for some people as they get older, they may also have more medical appointments already, so taking on a regimen of many medical appointments could seem—for some people—not wanting to, like, why do I want to go to the doctor’s office more than I already do?” *–FGD 1, Participant 1*	4		1				1					0					0			6
Study Fatigue	Feeling that there are too many research studies or too many flyers being handed out.	“There is a certain flyer fatigue in our generation.” *–FGD 1, Participant 1*	3		0				0					0					1			4

We divided our analysis into two major areas, barriers and facilitators to participation in research studies. Among barriers, five major themes were identified: “lack of knowledge about anal cancer”, “research focuses on anal cancer”, “stigma-related barriers”, “confidentiality concerns”, and “general barriers not specific to anal cancer”. Only one facilitatory theme was identified, “motivators to participation”.

Focus group discussions all began with open-ended, general questions about HPV and anal cancer (Additional file 3). These questions were designed to engage focus group participants into thinking about anal cancer. Questions yielded information regarding participants’ knowledge about HPV, anal cancer, and anal cancer screening and is presented in a separate manuscript (in preparation). We believe this information may also help to understand future participants’ motivation to enroll in similar studies.

We also identified an extensive list of ideas from participants that could be used to support recruitment (Additional file 4). A list of additions to our recruitment strategy that we implemented as a direct result of the FGDs is included as Table 4 which describes how they may be applicable to research focusing on different topics.

## Barriers (Table 3)

### Theme: lack of knowledge of HPV/Anal Cancer

A strong theme that emerged was that most participants lacked knowledge about anal cancer and screening for anal cancer. Many participants expressed that they had never heard about anal cancer until they encountered our clinic. There was agreement among most participants that they did not know who was at risk for anal cancer or which factors could increase their risk for anal cancer. Some men did not know that the risk of anal cancer was higher in MSM and in PLWH compared with the general population.

*“So it is higher in a gay man or is there, is a statistic[al variation between] gay men and straight men, …?” –FGD 2, Participant 2*

### Theme: Research Focuses on Anal Cancer

#### Sub-Theme: physical anal discomfort

Participants during each FGD felt that participating in a study on anal cancer and/or a study that included an anal exam presented unique barriers for recruitment. There was an expression of fear that the anal exam would cause physical discomfort from the HRA exam or psychological discomfort because the anus was involved. Participants describe fear surrounding having an anoscope–which is similar to a speculum used in vaginal examination–placed into his anus:

*“… that’s scary once it is clear what they are, like how that’s not that big, you know, that’s not a big deal to have something that big inside of you, the burning afterwards, it … doesn’t sound that appealing…” –FGD 1, Participant 1*

*“… I don’t think [men] will like it [the high-resolution anoscopy]. I can only speak from one side, I mean gay men, of course. Gay or straight men just don’t like fingers and things, some [men], up their butt. And get a man to go get that procedure done? Are you gonna get tested for it … nah. My partner is 71 years old, and since I started with this group, I told him about it and he would [say] like, no, no, no, no… and he finally came in. The look on his face when he came home… man. Yeah, I don’t think most men will like anyone looking up there…” –FGD 2, Participant 2*

#### Sub-Theme: Discomfort Talking about “Anal” or an anal exam

Our participants also expressed discomfort discussing a study that focused on anal cancer because they were not comfortable with the word “anal” and did not wish to discuss it with others in person or via social media. The word “anal” was associated with discomfort or embarrassment among both participants living with HIV and HIV-negative participants.

*“And I am pretty openly gay and HIV positive and everything, something like you said before about—it is kinda icky about anal.” –FGD 1, Participant 2*

#### Sub-Theme: Anus as a Center for pleasure

Regarding study recruitment at social venues (e.g., bars, nightclubs), some participants felt that they did not want to think about anal cancer or anal health while they were “…out to have a good time…” *–FGD 5, Participant 3*

*“If I was at the bar, I don’t want to think about all the consequences, my focus isn’t on health, and then there [are] other times [like] when I am in my doctor’s office, and I’m thinking a lot about health….” –FGD 3, Participant 3*.

### Theme: confidentiality

One barrier that came up in all FGDs was the fear that information shared or collected during screening would not be kept confidential. While this was brought up mostly by participants living with HIV, HIV-negative participants were quick to agree that they also had these concerns. Participants were concerned about what questions they would be asked during screening, who their information would be shared with, and what consequences there could be if their information was shared with primary care physicians or insurance companies.

*“I mean I am familiar with HIV studies and also thinking back to the 80’s when anonymity and confidentiality were—you know people were afraid of their providers having records that in some situations, sort of that the idea of preexisting conditions or the ability to sort of be stone-walled out of health coverage is certainly an experience within our memorable history; [I] did not trust that sort of integration.” –FGD 1, Participant 1*

Likewise, concerns about confidentiality also shaped respondents’ preferences for recruitment strategies. Some individuals did not want to risk being associated with an anal cancer study in public by picking up a flyer or by posting information about it on social media. These participants wanted their interest in anal cancer screening to be private.

*“I don’t consider myself sort of ‘out’ to the world, and I would decidedly not click on that [Facebook ‘share’ study material button]. I would look at it, try to memorize the number or something, but I would avoid that, just me, but I am part of the demographic. I would avoid that because I don’t want–it’s creepy how much Facebook tracks and all the other things, so I am more of a privacy oriented, I would never ‘like’ that and I wouldn’t want my friends to know that I liked that, again, just me.” –FGD 3, Participant 3*

### Theme: stigma-related barriers

#### Sub-Theme: Age Stigma

Our participants noted that in the gay community there is a stigma associated with aging. Participants said that they would be less likely to pick up recruitment material with photos of men who look older because they do not identify with pictures of people, particularly older men. Pictures of older men engaging in healthy activities, such as cooking, were specifically noted as being off-putting. Participants did not feel that these pictures represented them, even among participants that appeared to be older than the men depicted in recruitment images.

“They are nice, but they look a little old.” *–FGD 1, Participant 2*.

In addition, participants reported that that there is a belief that older people are not as attractive as their younger counterparts.

“He is still young enough to be, young and fit to be attractive for people to look at…” –*FGD 5, Participant 1*

Participants did recognize the dissonance in these thought processes. When asked to elaborate on this idea of not relating to images of people who look 50 + for the purposes of study recruitment–despite all participants being 50 + themselves–they felt that societal factors were at play.

*“The problem is that our culture is just so messed up about aging.” –FGD 1, Participant 4*

Participants said that stigma around aging may reduce desire to participate in a study that is targeted to “aging” men. It may also make them less likely to get screened or to worry about health issues that primarily concern older men.

#### Sub-Theme: General Cancer Stigma

There were also some expressions of stigma associated with cancer, no matter what type of cancer. This stigma was generational: participants expressed that in older generations, including their own, cancer was not discussed in public and carried some amount of stigma.

*“… it’s different enough because then the cancer thing is that the ‘C word’ is used.” –FGD 1, Participant 3*

#### Sub-Theme: other Stigma

Even in the San Francisco Bay Area in the current day, participants indicated that there is stigma associated with being gay, being out, and being a receptive partner (“bottom”).

*“How many people, do you think are radically in the closet about this stuff … I mean men that ha[ve] sex with men, they are really, are underground, about it.” –FGD 5, Participant 4*

*“Like there used to be an assumption that only bottoms get HIV. We have those biases and prejudices, they are not rational, and they are not true, but I think there is still some bottom shaming in our culture.” –FGD 1, Participant 3*

There was also shame and stigma associated with having anal cancer or an anal STI because of the location of the STI, likely because of the implication that it was associated with being a receptive partner.

*“Yeah, and I think for our generation, at least, I know through my life, sort of, anal STDs have more shame than say other STDs.” –FGD 1, Participant 1*

Taken together, these reflections hint at the significant role stigma plays on the lives of older MSM.

### Theme: General Barriers not specific to Anal Cancer

Other barriers included concerns over insurance coverage for the cost of visits, concerns about costs related to the study, concerns about the amount of time the study would require, and study fatigue, or feelings that there are too many research studies to join (Table 3).

**Table 4:** Additional recruitment efforts implemented as a result of qualitative study, grouped by theme or selected sub-theme, and suggestions of how these efforts could be applied into studies of other research topics

THEME OR SUB-THEME	ORIGINAL EFFORTS	ADDITIONAL EFFORTS IMPLEMENTED AS A RESULT OF FOCUS GROUP DISCUSSION	APPLICATION TO STUDIES OF OTHER RESEARCH TOPICS
**LACK OF KNOWLEDGE ABOUT HPV/ANAL CANCER**			
Lack of Knowledge of HPV/Anal Cancer	• ICF included information on anal HPV infection and anal cancer and groups at increased risk. • Physician discussed anal cancer screening with participants.	• Flyers/posters/website/ads contained statements to address knowledge gap, for example “Did you know that all men who have sex with men (top or bottom) are at risk for anal cancer?” • Created “HPV and Anal Cancer Education” presentation and presented it to community groups and health care provider groups in and around the San Francisco Bay Area.	• Include statements to address knowledge gaps specific to study topic on recruitment materials. • If large knowledge gap is present in target population, education campaign should be considered.
**RESEARCH FOCUSES ON ANAL CANCER**			
Physical Anal Discomfort	• Anal exam explained in detail at enrollment.	• Video created of principal physician describing the procedure in detail and showing all medical devices that will be used (speculum, swabs, colposcope). • Video available on website and presented in educational presentation.	• Study procedures should be thoroughly explained in an accessible way as part of recruitment materials and not just as part of the enrollment process.
**STIGMA-RELATED BARRIERS**			
Age Stigma	• Posters/flyers/website portrayed healthy men in older age groups.	• Eye catching “Peachy” campaign branded the AHHA Study with a peach emoji wearing a variety of underwear (Additional file 5). Posters/flyers/website/ads used new artwork. (The peach emoji is frequently used to symbolize a butt/bottom.)	• Researchers focusing on stigmatized populations, behaviors, or conditions should consider exploring these topics qualitatively before designing recruitment campaigns.
**CONFIDENTIALITY CONCERNS**			
Confidentiality	• Confidentiality of study participants addressed as part of protections for human subjects procedures.	• Developed smaller pocket-sized flyer and tear-offs on flyers. QR codes were also placed on all study materials that link to the study website so that participants do not need to take a flyer to have study information.	• Provide a variety of methods of obtaining study information that are both accessible and sensitive to privacy concerns.
**MOTIVATORS TO PARTICIPATION**			
Wanting Access to the Best Care	• Information on the UCSF ANCRE clinic’s experience and expertise included on clinic material.	• Included information on availability of anal cancer screening and treatment on posters/flyers/website/ads.	• Include important study institutions and collaborators on study recruitment materials.

FGD, Focus group discussion; ICF, Informed consent form; UCSF, University of California, San Francisco; ANCRE, Anal Cancer Center for Research and Education

**Table 5:** Facilitators identified from thematic analysis of focus group transcripts

THEME			Theme frequency by Focus Group Discussion (FGD)					
**Sub-Theme**	**Description**	**Example Quote**	**1**	**2**	**3**	**4**	**5**	**Totals**
**MOTIVATORS TO PARTICIPATION**								
Wanting to be Healthy	Wanting to live a healthy life and catch disease early through preventative care.	“I want to stay healthy and alive as long as I can. Somebody said “would you really like to live to be 100 [years old]?” I said yeah, if I could do it right.” *–FGD 5, Participant 5*	4	8	8	8	13	41
Altruism	Desire to help researchers or the community.	“… I think to just be part of these studies is part of how we are there for each other, how we sort of have each other’s back, like they are kind of reflecting this data with us and it’s going to help … the next generation.” *–FGD 3, Participant 2*	5	3	4	5	7	24
Money as a Facilitator/Motivator	Having a desire to join the study based upon financial compensation.	“Because I just firmly believe most people who are going to do a study, it’s because of the money factor.” *–FGD 4, Participant 4*	3	4	5	10	4	26
Wanting Access to the Best Care	Wanting to receive the best health care that is available.	“If I have a group that is specific about gay, anal cancer where am I going to get better care than that? So, if it affords me the opportunity to get screened by the best of the best should I find out that I have something, which was my case, then I have the comfortable position of knowing I have seven years of care [for the ANCHOR study]. It’s like this is the best, sure I’ll drive down from Sonoma every six months.” *–FGD 4, Participant 5*	3	1	1	5	3	13
Wanting Health Education about the Anal Exam	Desire to be educated or obtain an understanding of the anal exam as it relates to their health.	“I’ll speak from my motivation to do which is if I have access to free schooling ongoing for seven years, in the case of the ANCHOR study I believe, that’s a huge value to me.” *–FGD 4, Participant 5*	6	8	2	4	3	23
Want Recommendation from their Provider	Wanting their medical provider to recommend HPV or anal cancer screening.	“So, if you can actually if the doctor himself or herself actually mentioned it, but that is probably a lot better than just having the information.” *–FGD 2, Participant 1*	2	4	0	1	0	7
Gay Provider	Wanting a gay provider for health care needs including the anal exam.	“I had one gay doctor and I am sure that he … if he wasn’t up to speed with it he could have gotten up to speed. The other docs, you know, I don’t have a whole lot of perception that they are familiar, or they are as comfortable with gay men’s health as I’d like them to be with mine.” *–FGD 1, Participant 1*	3	1	0	1	0	5

## Facilitators (table 5)

### Theme: motivators to participation

#### Sub-Theme: Altruism

One of the most discussed motivators for study participation was altruism. Most participants acknowledged that along with other facilitators, they or people in our target population would likely have a strong motivation to help others in their community. Participants thought that this would be particularly important to older MSM who experienced the HIV/AIDS epidemic in the 1980’s-1990’s.

*“I think there are enough people that—and for me it would be a willingness to share on my timeline. To say this is something important to people of my age, and I have a lot of friends that I have known since the 80’s that we have all had HIV.” –FGD 1, Participant 1*

*“Yes, I am going, and I think to just be part of the study is part of how we are there for each other, how we sort of have each other’s back like we are kind of reflecting this data with us, and it’s going to help the next generation.” –FGD 3, Participant 2*

#### Sub-Theme: wanting to be Health and Wanting Access to the best care

In every FGD, multiple participants mentioned a desire to be healthy, to catch and treat diseases early, and to receive preventative care as motivators to be part of our study and other studies. Other personal benefits mentioned by many participants were money received as an incentive to participate, wanting to receive the best quality of care, and wanting to have ongoing health education about the anal exam and their health.

*“And I think a portion of men who have sex with men are interested in prolonging their vital and healthy years.” –FGD1, Participant 4*

#### Sub-Theme: other motivators

Two motivators related to patient-provider communication were mentioned only a few times in the FGDs but may be important. The first is having a direct recommendation to join the study by a clinical provider. Participants felt that they would be more likely to join studies such as ours if their provider recommended the study.

*“So, if you can actually, if the doctor himself or herself actually mentioned it, but that is probably a lot better than just having the information.” –FGD 2, Participant 1*

Participants also discussed the advantages of having a doctor who was gay. They felt they would be better understood by someone who was similar to them in this regard, and they felt that they would be more comfortable discussing their health needs with a gay provider. This sentiment is understandable when studies continue to show inadequacies in LGBT competency training among physicians and other health care professionals, with associated disparities in clinical competency [20, 21].

## Discussion

The primary goal of this study was to identify new and actionable methods to increase recruitment into the parent study, the AHHA Study. We modified our recruitment strategy and recruitment materials in accordance with the feedback received from our focus group participants and increased recruitment as a result–increased from 2 to 3 participants per month to 9–10 per month (Table 4).

Through thematic analysis of the FGDs, we were able to uncover several themes that may influence participation in studies conducted among older MSM, particularly older MSMLWH. As we enter a new frontier with a large, aging, population of PLWH, more research will be needed to identify screening, treatment, and preventative interventions that target this demographic. Finding effective methods to recruit members of this population into studies is, therefore, crucial and may require substantial creativity and thoughtfulness of study teams.

### Barriers to participation

Many of the barriers to recruitment that we identified in our study may be unique to studies of anal cancer or other anal health topics. For example, participants expressed discomfort with most things ‘anal’. There was fear about the anal exam, discomfort with the word ‘anal’, and discomfort with associating a pleasure center with disease. However, some of these barriers may be extrapolated to studies of other sensitive topics. Studies on barriers to cervical cancer screening show the same link between fear of discomfort and willingness to be screened [22, 23]. Just as it was difficult to motivate individuals during the early era of cervical cancer screening to be screened, these barriers may be difficult to surmount for anal cancer screenings until the topic of anal cancer has been more normalized in our culture. Health education and open discussion with medical providers about men’s anal health, but also women’s anal health–as women are also at risk of anal cancer–may begin to shift this perception in our culture. Celebrities such as Farrah Fawcett, Marcia Cross, and Michael Douglas continue to help to build awareness and normalize HPV-associated cancers.

Accordingly, we found a surprising lack of awareness about anal cancer in our participants. Most of the men who had heard of anal cancer learned about it through contact with the UCSF ANCRE clinic and/or associated anal cancer studies. Even among those men, there was an important lack of knowledge about what causes anal cancer and who is at risk. In our study, participants thought that men in their communities believed that only “bottoms” (receptive partners) were at risk for anal cancer, or that only those who had HIV were at risk. Men indicated that if they were HIV-negative and they saw HIV on a flyer they would disregard it. This is an important barrier to recruitment for our study, and potentially for other studies where it is not common knowledge which groups are at higher risk of developing the disease. Individuals may be less willing to participate if they do not believe themselves to be impacted by the illness under study.

These findings prompted us to add wording to our recruitment materials that all gay men are at risk for anal cancer, as well as to remove mentions of HIV in our recruitment materials (Additional file 5). Other studies of men living with HIV and HIV-negative men should consider removing mention of HIV from materials rather than highlighting that both HIV statuses are invited to participate.

Another important barrier more specific to our population was confidentiality. Many of our participants are men who experienced some of the unsettling aspects of the HIV epidemic in the 1980 and 1990 s. Participants shared issues of loss of confidentiality and stigma about being gay or living with HIV that were related to studies that took place during the early parts of the HIV epidemic. There is a cultural history among older PLWH that may not be present or problematic in studies that are not related to HIV/AIDS. Researchers should not only consider confidentiality within their study, but also how the desire for privacy may interfere with study recruitment or participation. We found that participants may not even pick up a flyer if they feel that the flyer may breach a confidence to someone who sees it. The AHHA Study responded to this concern by using smaller pocket-sized flyers, tear-offs on flyers, and QR codes [24] that link to the study website. Also, while other studies may have success using social media for recruitment, many men in our target population did not feel comfortable forwarding the study Facebook site to others because of privacy and confidentiality concerns.

A concerning finding was that despite growing acceptance of, and openness within, MSM communities, participants noted that there was stigma associated with being gay, particularly with being a receptive partner. These findings reinforce the presence of a culture of stigma against receptive partners also known as “bottom shaming” as described elsewhere [25–27]. Additionally, our study and others [25] have found that shame associated with having an anal STI or anal HPV/anal cancer was more profound than that for similar STIs that were located genitally or orally. This shame will obviously reduce participation in studies focusing on anal health and may also limit individuals’ willingness to receiving screening or treatment for any kind of anal disease. Again, this may be addressed by normalizing receptive anal sex as one of many choices available to all individuals, by educating that anal HPV associate disease can occur even in those who do not participate in receptive anal sex, and by promoting healthy sexual behaviors, and promoting prevention–including screenings, treatments, and vaccinations.

Another stigma that was highlighted in our analysis was stigma in the MSM community associated with aging. While it would be beneficial for the public health community to target negative perceptions associated with age in this community and in society at large, researchers may need to be more creative until this pervasive attitude shifts. Using pictures of older individuals may alienate–instead of attract–older MSM. The AHHA Study shifted from photographs to a cartoon of a peach–an emoji frequently used to symbolize a butt/bottom–for its flyers (Additional file 5) and has had more success with recruitment from these materials [28].

### Facilitators

Consistent with other studies [29–32] altruism was a strong motivator for study participation. The general desire to be healthy, have access to preventative care, know about one’s health status, and be able to receive the best health care possible were also important motivators and consistent with other findings [13, 22]. Future studies of any topic could craft recruitment messages tailored to better highlight these positive, altruistic aspects of study participation. Our participants wanted recommendations from health care providers to participate in research and expressed a desire for gay providers when possible. Future researchers focusing on PLWH or MSM would benefit from developing recruitment strategies to educate local providers in the needs of PLWH and HIV-negative MSM and may improve recruitment through referrals. For research not related to PLWH, researchers could consider linkages with health care providers early in study design to aid in recruitment.

### Limitations

Because we recruited participants from the ANCRE clinic in San Francisco and among men connected with the clinic through their networks, men who were willing to participate in our focus groups may have been more educated on anal cancer and screening than other members of our target population. This may limit our ability to understand facilitators and barriers associated with less connected members of our target population. However, this makes our findings on lack of knowledge particularly noteworthy as aging MSM populations not connected with anal cancer clinics or clinicians may have even less understanding of anal HPV/cancer, and by association even less willingness to participate, than our participants. Another limitation is that we did not include women in this study. The primary reason that we conducted this study was to aid in recruitment for the AHHA Study, which only included MSM because of their relatively high risk for developing anal cancer. The ANCRE clinic treats and conducts research on all genders, and a study of recruitment of older women would surely provide additional insight into this topic.

## Conclusion

Determining how to successfully recruit older MSMLWH and HIV-negative MSM into health studies will be increasingly important if we are to continue to meet the health needs of aging PLWH. For this population, researchers need to be aware of barriers to participation including feelings of discomfort with discussion of certain topics. Confidentiality and privacy concerns are of particular importance in this population and should also be considered. Researchers also need to keep in mind that stigma continues to play a significant role in influencing older MSM to participate in studies. Factors that motivate study participation may be capitalized on, including altruism and individuals’ desires to be healthy by receiving preventative care and efforts to receive the best care possible.

The themes that we identified as barriers to participation are most closely applicable to research that targets older MSMLWH or HIV-negative MSM as well as to research that focuses on anal HPV, anal cancer, or other health conditions involving the anus. However, many themes can be extrapolated to other population including addressing knowledge gaps, explaining study procedures in an accessible manner before enrollment, providing privacy in acquiring study information, and accounting for stigma during recruitment.

## Electronic supplementary material

Below is the link to the electronic supplementary material.

Supplementary Material 1

## Data Availability

Data will be available upon request. Please contact Alexandra Hernandez (alexandra.hernandez@ucsf.edu) to request access.

## Code availability

Focus group code book and Dedoose coding document are available from corresponding author upon request.

## List of abbreviations

**ART**: Antiretroviral therapy.
**ANCRE**: Anal Neoplasia Clinic, Research and Education Center.
**CD4**: Cluster of differentiation 4.
**COREQ**: COnsolidated criteria for REporting Qualitative research.
**FG/FGD/FGDs**: Focus group discussion(s).
**HIV/AIDS**: Human immunodeficiency virus/acquired immunodeficiency syndrome.
**HPV**: Human papillomavirus.
**HRA**: high resolution anoscopy.
**HSIL**: High-grade squamous intraepithelial lesion.
**LGBT**: lesbian, gay, bisexual, transgender.
**MSM**: Men who have sex with men.
**MSMLWH**: Men who have sex with men living with HIV.
**PLWH**: People living with HIV.
**QR code**: Quick response code.
**UCSF**: University of California, San Francisco.
**AHHA Study**: Anal HPV.
**HIV**: and Aging Study.
