# Supplementary material for: “The problem is that our culture is just so messed up about aging.” Recruiting older men who have sex with men (MSM) into research studies: an example from a study of aging, HIV, and anal HPV
Source: BMC Med Res Methodol. 2022 Nov 18;22:296. doi: 10.1186/s12874-022-01752-0 (PMC9675202; doi:10.1186/s12874-022-01752-0)
Supplement: Supplementary file 1 — Supplementary Material 1 [file 12874_2022_1752_MOESM1_ESM.docx]

**Additional file 1: COREQ: Consolidated criteria for reporting qualitative research: a 32-item checklist for interviews and focus groups [14]**

| **Item #** | **Guide Questions/Description** | **Reported on Page #** |
| --- | --- | --- |
| **Domain 1: Research team and reﬂexivity** | | |
| *Personal Characteristics* | | |
| 1. Interviewer/facilitator | Which author/s conducted the interview or focus group? | 30 |
| 2. Credentials | What were the researcher’s credentials? e.g. PhD, MD | 30 |
| 3. Occupation | What was their occupation at the time of the study? | 30 |
| 4. Gender | Was the researcher male or female? | 30 |
| 5. Experience and training | What experience or training did the researcher have? | 30 |
| *Relationship with participants* | | |
| 6. Relationship established | Was a relationship established prior to study commencement? | 8 |
| 7. Participant knowledge of the interviewer | What did the participants know about the researcher? e.g. personal goals, reasons for doing the research | 8 |
| 8. Interviewer characteristics | What characteristics were reported about the interviewer/facilitator? e.g. bias, assumptions, reasons and interests in the research topic | 8 |
| **Domain 2: Study design** | | |
| *Theoretical framework* | | |
| 9. Methodological orientation and Theory | What methodological orientation was stated to underpin the study? e.g. grounded theory, discourse analysis, ethnography, phenomenology, content analysis | 8 |
| *Participant selection* | | |
| 10. Sampling | How were participants selected? e.g. purposive, convenience, consecutive, snowball | 5 |
| 11. Method of approach | How were participants approached? e.g. face-to-face, telephone, mail, email | 5 |
| 12. Sample size | How many participants were in the study? | 9, ­­­Table 2 ­(31) |
| 13. Non-participation | How many people refused to participate or dropped out? Reasons? | 6 |
| *Setting* | | |
| 14. Setting of data collection | Where was the data collected? e.g. home, clinic, workplace | 7 |
| 15. Presence of non-participants | Was anyone else present besides the participants and researchers? | 7-8 |
| 16. Description of sample | What are the important characteristics of the sample? e.g. demographic data, date | 4-5, ­­­Table 1 ­(30) |
| *Data collection* | | |
| 17. Interview guide | Were questions, prompts, guides provided by the authors? Was it pilot tested? | 7 |
| 18. Repeat interviews | Were repeat interviews carried out? If yes, how many? | 7 |
| 19. Audio/visual recording | Did the research use audio or visual recording to collect the data? | 7 |
| 20. Field notes | Were ﬁeld notes made during and/or after the interview or focus group? | 7 |
| 21. Duration | What was the duration of the interviews or focus group? | 7 |
| 22. Data saturation | Was data saturation discussed? | 9 |
| 23. Transcripts returned | Were transcripts returned to participants for comment and/or correction? | 9 |
| **Domain 3: Analysis and ﬁndings** | | |
| *Data analysis* | | |
| 24. Number of data coders | How many data coders coded the data? | 9 |
| 25. Description of the coding tree | Did authors provide a description of the coding tree? | N/A |
| 26. Derivation of themes | Were themes identiﬁed in advance or derived from the data? | 9 |
| 27. Software | What software, if applicable, was used to manage the data? | 9 |
| 28. Participant checking | Did participants provide feedback on the ﬁndings? | 5, 20 |
| *Reporting* | | |
| 29. Quotations presented | Were participant quotations presented to illustrate the themes/ﬁndings? Was each quotation identiﬁed? e.g. participant number | 11-20 |
| 30. Data and ﬁndings consistent | Was there consistency between the data presented and the ﬁndings? | 21-25 |
| 31. Clarity of major themes | Were major themes clearly presented in the ﬁndings? | 10, Table 3 (32-34) |
| 32. Clarity of minor themes | Is there a description of diverse cases or discussion of minor themes? | 11-20 |

**Additional file 2: Focus group discussion (FGD) script read at the beginning of each FGD by main facilitator**

| **Welcome**  Hello, my name is {*insert name of facilitator 1}* and this is *{name of facilitator 2*} and we will be leading the focus group today. We both work at UCSF in the ANCRE Clinic, which stands for Anal Neoplasia Center for Research and Education. We invited you here today to take part in a focus group about one of our studies. You were selected because of your interest in our study population as well as in the subject matter.  The UCSF ANCRE Clinic is starting a new study called the AHHA Study, or the Anal HPV, HIV, and Aging Study. The goal of this study is to examine how normal aging, HIV infection, and HPV infection interact to promote anal cancer precursors, or anal cancer. We know that each of these factors increase the risk of anal cancer individually, but we don’t know if the risk is increased if all three factors (aging, HIV, and HPV) occur together. To address our research questions, the AHHA study will recruit men and trans-people who have sex with men, both HIV-positive and HIV-negative, who are over 50 years old. We hope to recruit about 1300 men for our initial visit, equally divided into three age groups (50-59, 60-69, and 70+).  At each study visit, participants in the AHHA Study will complete a behavioral questionnaire, a grip strength test, a walk test, and give blood samples to test for HIV-related variables and markers of biological aging. The doctor will also take an anal swab for anal HPV infection testing and perform an anal clinical examination called high resolution anoscopy, a procedure very similar to a Pap smear for women. The exam is rarely painful, but can be a bit uncomfortable.  Does anyone have any questions about the AHHA Study? {*pause and answer questions}*  Great. Also, I will be available after the focus group to answer any questions about the study or anal HPV and anal cancer.  The purpose of this focus group is to learn more about where to find eligible men and trans-people for our study. We are also interested in your thoughts regarding possible barriers to participation, as well as what might motivate our target population to join our study.  Before we begin, we are going to review a few standard guidelines to help facilitate a warm and friendly environment.  **Guidelines**   - First, we will audio-record today’s focus group to ensure we don’t miss any of your valuable comments. You may be assured of complete confidentiality. Only the study team will listen to this recording. Does anyone object to being recorded today? {*Pause and excuse anyone who does not consent to being recorded*} - Since we’re recording, it’s best if only one person speaks at a time, otherwise it’s difficult to understand the recording. - We are on a first name basis. - There are no right or wrong answers, only differing points of views. You don’t need to agree with others, but please listen respectfully as others share their views. - Please feel free to share your point of view even if it differs from what others have said. Keep in mind that we’re just as interested in negative comments as positive comments, and at times the negative comments are the most helpful. - We ask that you turn off or silence your cell phones. If you cannot and if you must respond to a call, please do so as quietly as possible and rejoin us as quickly as you can. - My role as moderator will be to guide the discussion. - To respect your time, we will either finish before or end at {insert time} but we will not go over. - Can you think of any other guidelines you’d like to share?   Does anyone have any questions about the focus group before we begin? {*Pause and answer questions}* |
| --- |

**Additional file 3: Focus group discussion guide**

| **Please note, for all responses, we may ask you to elaborate or we may have follow-up questions to learn more about perceived issues, experiences, and feelings.**   1. Have you ever heard of anal cancer? What comes to mind when you think of anal cancer? 2. Our study targets men and transgender people who have sex with men and who are over the age of 50 years. Do you think that this population would be interested in our study? 3. How do you think men and trans-people in our target age group would react to…    1. the anal exams that we described?    2. the anal exam and some other procedures are considered standard of care and participants insurance will be billed for these procedures?    3. they will receive a $50 stipend? 4. What kinds of things do you think might motivate people to participate in this study?   {*If they don’t spontaneously mention things like altruism, money, concern about their health and/or anal cancer, HIV survivors/thrivers, and other common motivations then suggest these as possible motivations and see what they think*.}   1. How and where would you suggest recruiting men and transgender people who have sex with men who are ***HIV-positive*** for participation in this study? 2. How and where would you suggest recruiting men and transgender people who have sex with men who are ***HIV-negative*** for participation in this study? 3. How and where would you suggest recruiting men and transgender people who have sex with men who are ***50-59? 60-69? 70+*** for participation in this study?   {For 5-7, *If they don’t spontaneously mention community-based organizations and/or other businesses and community leaders, then suggest these as possible sources of recruitment and see what they think*.}   - 1. *A follow up question could be*: Who do you know who would be willing to refer folks to us? For example, consider people who are medical/health professionals, case workers, Community-Based Organizations (CBO’s), spiritual leaders, educators, sports groups, etc.   2. *A follow up question could be*: Do you know any very well-known public figures who could help disseminate information about the AHHA study?   3. *A follow up question could be*: How do you usually find/learn out about local events for MSM and trans-people? {*examples might include: LGBT newspapers, radio, internet, social media, gossip, friends, church, other social groups}*   4. How do you feel about a buddy system for enrollment?  1. Once someone has signed up for the study, what kinds of things could we do to encourage people to stay in this 3-year study? 2. Do you have any other thoughts about recruitment and retention for this study that you’d like to share now? |
| --- |
| These questions were used to guide the discussion. Not all questions were asked to all FGD participants and question may have been slightly modified. Follow-up questions may have been generated during discussion. |

**Additional file 4: Recruitment ideas proposed during focus group discussions. Names have been deidentified to preserve confidentiality**

| **Focus Group Discussion proposed Recruitment Ideas** | |
| --- | --- |
| Members of the Community | - Head of local gay chorus - Head of the local gay history museum - Head of local support group - Local drag queens - Local movie star - Well-known activist - Mayoral candidate - Well-known LGBT-friendly politician - Local porn star - Well-known drag queens - Local business leader - Local business owner - Non-profit executive - Community participant ambassadors |
| Community Groups/Foundations | - San Francisco AIDS Foundation - Bridgeman group - Elizabeth Taylor 50+ network - Pacific Center for Human Growth - LGBTQ Centers   - Billy DeFrank Center - Aging and gay men’s health site/camp - Senior Lunch Program - OurTownSF - Senior Housing   - Openhouse SF   - Senior Housing in Santa Rosa - East Bay AIDS Center - Eureka Recreation Center - Gay Alcoholics Anonymous Meetings - Castro Country Club - California Men’s Gathering - San Francisco Fire Department   - Fireman Calendar - Gear Up Weekend - Black Leather Wings - Gay Chorus |
| Physical Advertisements | - BAR Magazine - AARP Magazine - POZ Magazine - Out Magazine - East Bay Express - Sponsor for advertisements   - - Alcohol companies - Advertisements on buses, inside buses, and on bus shelters |
| e-Advertisements | - Focus group website advertisements - SurveyMonkey website ads - AARP website advertisements - Craigslist website advertisements |
| Facebook | - Facebook advertisements - Events organized via Facebook platform - Asking organizations and individuals to like/share/post about the study - Ask people with many followers to post/re-post/re-tweet |
| Instagram | - Instagram advertisements |
| Dating/“Hookup” apps | - Grindr - OkCupid - GROWLr - Scruff - Adam4Adam - Daddyhunt - Squirt.org - BarebackRT.com - Asspig.com - AdultFriendFinder - SilverDaddies - Manhunt - OnlineBootyCall - dudesnude - Hornet |
| YouTube video | - Explaining exam - Celebrity having anal exam or discussing anal exam - Using a cartoon to explain anal exam |
| Education Campaigns | - Anal Cancer awareness day - Awareness campaign with sports teams - HPV education awareness |
| Event Ideas | - Give away donut holes “Hole in health care screening”   - - Fundraiser held at local community gathering location or bar     - Advertisements on screens at local sports bar     - Event with dancing   - ‘Prime’ dance party (men in their ‘prime’ and admirers) - Trivia night |
| Give Aways | - Temporary tattoos - Rainbow bracelets - Condoms - Gift certificates for use at community businesses - Gift certificates to Castro stores |
| HIV+ Healthcare Sites | - Shanti Clinic - UCSF Alliance Health Project - STD Clinic - Ward 86 - Trans Health Clinic in Sacramento - Kaiser HIV Community Camp - Summit Hospital - SF City Clinic - San Francisco AIDS Foundation   - Strut/Magnet Clinic |
| Community Gathering | - Bars   - Bars through Bay Area   - Bars in common vacation destinations (ex. Guerneville, Rushen River Valley) - Bathhouses - Local Community Potluck - Groundswell Community and Retreat Center - Gyms - Hair Salons - Leather stores - Vitamin stores - Coffee Shops - 55 Laguna Senior Housing - Pharmacies serving HIV+ populations - Grocery stores in the Castro - Harvey Milk Plaza |
| Pets | - Pets Are Wonderful Support (PAWS) organization - Pet grooming salons - Dog Parks   - Duboce Park Dog Play Area |
| Religious Groups | - Friends Church - Metropolitan Community Church - Most Holy Redeemer Catholic Church - Sha'ar Zahav Jewish Synagogue - Trinity Church SF - The Church of Saint John the Devine - Saint Cyprian's Episcopal Church - Saint Agnes Catholic Church - San Domingo, Healing Waters - Allen Temple |
| Flyer Language | - Puns involving “butt”   - “When was the last time your doctor touched your butt?”   - “Make sure that your butt is covered”   - “We like old butts”   - “Your butt is on the line” - Puns involving “hole”   - “Is there a hole in your healthcare?”   - “The hole in a gay man’s healthcare is he is not getting screened for anal cancer” - “Cover your ass” - “Have an AHHA Moment!” |
| Other Flyer Ideas | - Give out condoms with flyers - Different images on flyers   - Images of a leather daddy |
| Buddy Screening | - Pair up with someone and make promise to get screened |
| Retention | - Thank you cards |

**Additional file 5: Flyers used for AHHA study recruitment before (left) and after (right) input from focus group discussions**

**
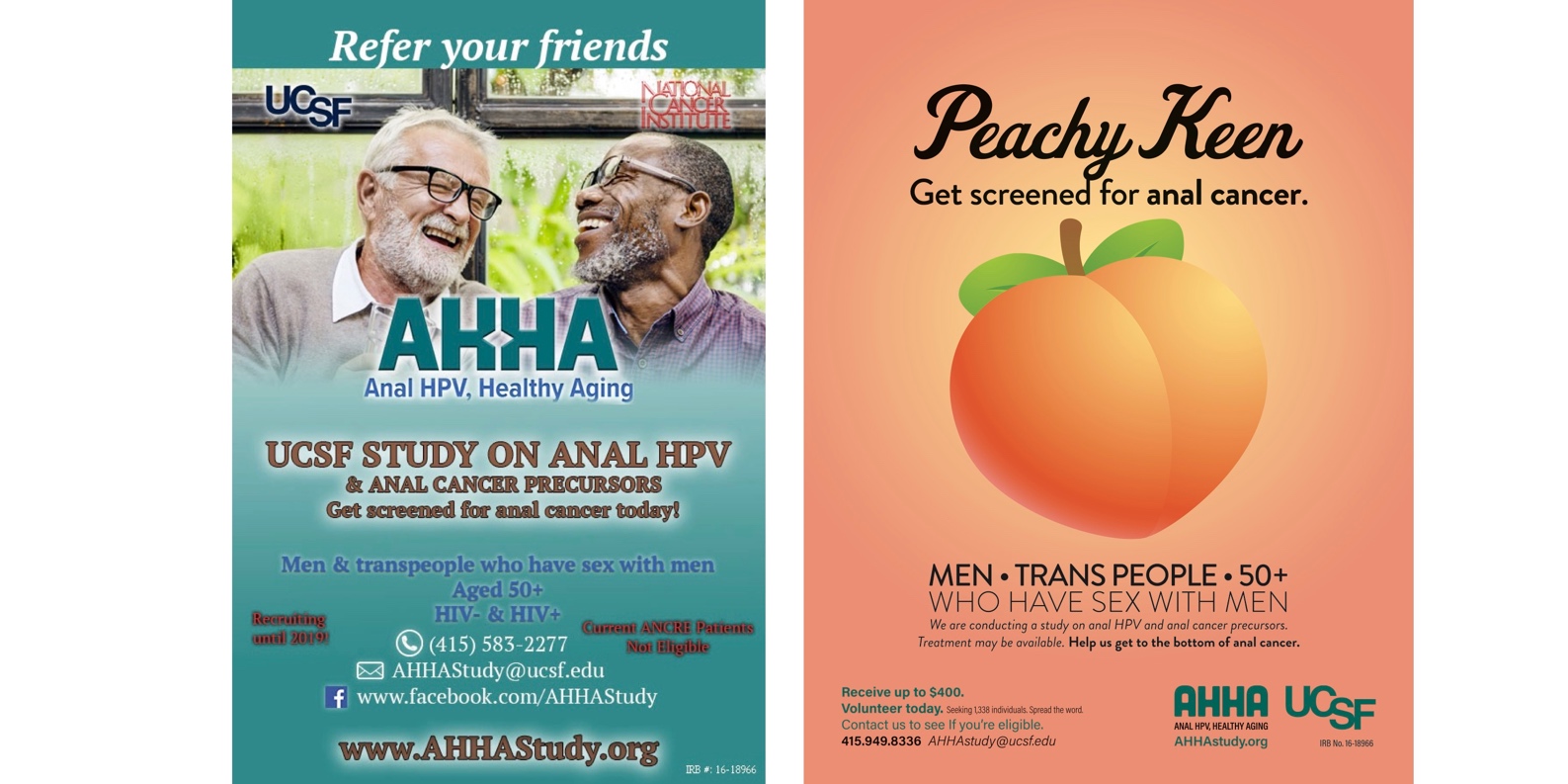
**
